# Effect of a High-Starch or a High-Fat Diet on the Milk Performance, Apparent Nutrient Digestibility, Hindgut Fermentation Parameters and Microbiota of Lactating Cows

**DOI:** 10.3390/ani13152508

**Published:** 2023-08-03

**Authors:** Suran Liu, Ziwei Wei, Ming Deng, Zhenyu Xian, Dewu Liu, Guangbin Liu, Yaokun Li, Baoli Sun, Yongqing Guo

**Affiliations:** 1 Guangdong Laboratory for Lingnan Modern Agriculture, South China Agricultural University, Guangzhou 510642, China; abc1452118779@126.com (S.L.); 13721016659@163.com (Z.W.); dengming@scau.edu.cn (M.D.); z840432229@163.com (Z.X.); dwliu@scau.edu.cn (D.L.); gbliu@scau.edu.cn (G.L.); ykli@scau.edu.cn (Y.L.); 2 College of Animal Science, South China Agricultural University, Guangzhou 510642, China; 3 Fuyang Bright Ecological Wisdom Ranch, Bright Dairy & Food Co., Ltd., Fuyang 236328, China

**Keywords:** high-starch diet, high-fat diet, milk performance, apparent digestibility, hindgut flora

## Abstract

**Simple Summary:**

Fat content is critical to milk quality, health and economic value. Fat content can be affected by diet composition. Diet-induced milk fat depression (MFD) is a specific reduction in milk fat synthesis caused by feeding cows high contents of rapidly fermentable carbohydrates or polyunsaturated fatty acid (PUFA). However, the mechanisms involved in MFD have not been fully elucidated and substantial controversy remains. Therefore, the aim of this study was to provide reference data for exploration of the relationship between milk fat synthesis and nutrient digestion as well as hindgut microbials.

**Abstract:**

In this study, changes in milk performance, nutrient digestibility, hindgut fermentation parameters and microflora were observed by inducing milk fat depression (MFD) in dairy cows fed with a high-starch or a high-fat diet. Eight Holstein cows were paired in a completely randomized cross-over design within two 35 d periods (18 d control period and 17d induction period). During the control period, all cows were fed the low-starch and low-fat diet (CON), and at the induction period, four of the cows were fed a high-starch diet with crushed wheat (IS), and the other cows were fed a high-fat diet with sunflower fat (IO). The results showed that, compared to when the cows were fed the CON diet, when cows were fed the IS or IO diet, they had lower milk fat concentrations, energy corrected milk, 3.5% fat-corrected milk yield, feed efficiency and apparent digestibility of NDF and ADF. However, cows fed the IO diet had a lower apparent digestibility of ether extracts. In addition, we observed that when cows were fed the high-starch (IS) or high-fat (IO) diet, they had a higher fecal concentration of propionate and acetate, and a lower NH_3_-N. Compared to when the cows were fed the CON diet, cows fed the IS diet had a lower pH, and cows fed the IO diet had a lower concentration of valerate in feces. In the hindgut microbiota, the relative abundance of Oscillospiraceae_UCG-005 was increased, while the Verrucomicrobiota and Lachnospiraceae_AC2044_group were decreased when cows were fed the IO diet. The relative abundance of Prevotellaceae_UCG-003 was increased, while the Alistipes and Verrucomicrobiota decreased, and the Treponema, Spirochaetota and Lachnospiraceae_AC2044_group showed a decreasing trend when cows were fed the IS diet. In summary, this study suggested that high-starch or high-fat feeding could induce MFD in dairy cows, and the high-fat diet had the greatest effect on milk fat; the high-starch or high-fat diet affected hindgut fermentation and apparent fiber digestibility. The changes in hindgut flora suggested that hindgut microbiota may be associated with MFD in cows.

## 1. Implications

Fat content is critical to milk quality, health and economic value. In addition, fat content can be affected by the components of the diet. Diet-induced milk fat depression (MFD) is a specific reduction in milk fat synthesis caused by feeding with high contents of rapidly fermentable carbohydrates and polyunsaturated fatty acid (PUFA). However, the mechanisms involved in the MFD have not been fully elucidated and substantial controversy remains. Therefore, the aim of this study was to provide data to explore the relationship between milk fat synthesis, nutrient digestion and hindgut microbials.

## 2. Introduction

The unique nutritional value of milk can be attributed to the presence of short-chain fatty acids and medium-chain fatty acids which are important sources of energy for human internal organs [1]. However, achieving high levels of milk yield and maintaining optimal milk fat synthesis remains a challenge in the current dairy production system [2]. Milk fat depression (MFD) in dairy cows is a reduction in milk fat concentration and yield, with no change in milk yield or other milk components [3]. At present, the nutritional factors causing MFD in dairy cows usually includes two diets, one is a diet rich in rapidly fermenting carbohydrates, low physically available fiber, or both, and the second is a diet supplemented with unsaturated fatty acids (UFA), especially marine lipids containing eicosapentaenoic acid (EPA, 20:5N-3) and docosahexaenoic acid (DHA, 22:6N-3) [4].

Since the discovery of MFD, scholars have continuously explored the mechanism of MFD and have put forward four main theories: acetate and β-hydroxybutyric acid (BHBA) deficiency [5,6], insulin–glucose release theory [7], trans fatty acid inhibition theory [8], and biohydrogenation theory. Among them, the biohydrogenation theory has been widely studied and accepted in recent years, and the reduction in milk fat synthesis is attributed to reduced mammary capacity for lipid synthesis caused by bioactive trans FA intermediates generated during altered rumen biohydrogenation of unsaturated FA by rumen microbes [9]. However, the above views mainly focus on the rumen trans fatty acids or flora, and studies have shown that the hindgut plays a important role in milk production and health [10]. For example, when large amounts of starch are fermented in the hindgut, which could increase the contribution of the concentrate-rich diet to both energy supply and health issues [11].

In addition, studies have shown that changes in diet composition can lead to changes in the microflora of the gastrointestinal tract of cows, resulting in changes in nutrient digestibility and intestinal function [12,13]. We therefore hypothesised that high-starch or high-fat diet induced MFD can alter hindgut fermentation and microbiota in cows, but the effects of these two models differed. In this study, we used traditional animal nutrition research techniques and 16S rRNA high-throughput sequencing to investigate and compare the effects of two dietary patterns on nutrient digestion, hindgut fermentation and microbiota in dairy cows.

## 3. Materials and Methods

### 3.1. Management of Cows

The experiment was conducted on the Wenshi dairy farm in Zhaoqing, Guangdong, China. All cows and experimental protocols in this study were reviewed and approved by the Animal Care and Use Committee of the South China Agricultural University, Guangzhou, China (approval number SCAU#2013-10). Eight Holstein dairy cows with an average body weight (BW) of 566 ± 39 kg and days in milk (DIM) of 91 ± 16 days (mean ± SD) were selected. The cows were housed individually in a separate enclosure (2.5 m × 2.5 m) with rubber mattresses throughout the study. All cows had constant ad libitum access to fresh drinking water and were fed twice daily at 0700 and 1800 h with a total mixed ration (TMR), allowing for at least 5–10% residual (as-fed basis) feed. The cows were milked at 0630 and 1730 h. The experimental diets were designed according to feeding standards [14], and details can be seen in Table 1.

### 3.2. Experimental Design and Treatments

This trial was a completely randomized design with eight Holstein cows divided into Groups A and B having four head per group. The experiment was divided into a pre-experiment period (18 d), two induction periods (34 d) and one washout period (18 d), details are shown in Table 2. There were three dietary treatments, two of which may induce milk fat depression in dairy cows: (1) a basic total mixed ration (CON); (2) a high-starch diet with fine ground wheat (IS, pass through 1.5 mm sieve); and (3) a high-fat diet with sunflower fat (IO). As shown in Table 2, in the pre-experiment, 8 cows were on the CON diet; in period 1, 8 cows were randomly assigned to the IS or IO diet, with 4 cows in each treatment diet. In period 2, the cows that were fed IO or IS in period 1 were switched to the other treatment diet in period 3.

### 3.3. Feed Intake, Apparent Nutrient Digestibility

The diets and orts amount of each cow were recorded daily and put in 65 °C for 48 h to calculate dry matter intake (DMI). The diets and orts samples were collected from each cow during the last 3 d of the pre-experiment period, and periods 1 and 3. Diets were dried at 65 °C for 48 h, and the samples were ground using a Wiley (A.H.TIOmas, Philadelphia, PA, USA) mill (1 mm sieve). The dry matter (DM), organic matter (OM), crude protein (CP) (Method: 98903), starch (Method: 948.03), ash (Method: 942.05) and ether extract (EE) (Method: 2003.05) were determined based on the Association of Official Analytical Chemists method (AOAC, 1990). Determination of neutral detergent fiber (NDF) and acid detergent fiber (ADF) in diets were performed using the traditional method [15]. Determination of acid insoluble ash (AIA) in diets and feces were performed using a previous method [16].

Fresh fecal samples were collected by rectal palpation on 15 d (04:00, 09:00, 14:00, 19:00), 16 d (05:00, 10:00, 15:00, 20:00) and 17 d (06:00, 11:00, 17:00, 22:00) of pre- experiment period, and periods 1 and 3. The 3 d feces sample from each cow was mixed to 400 g, added to 10% tartaric acid at one-quarter fecal weight, dried at 65 °C for 48 h, ground using a Wiley (A.H.TIOmas, Philadelphia, PA, USA) mill and passed through a 1 mm sieve. Determination of nutrients in fecal samples was similar to the procedure for diets. Apparent nutrient digestibility was calculated using the formula [17]: D = [1 − (Ad × Nf)/(Af × Nd)] × 100, where Ad (g/kg) and Af (g/kg) represent the AIA in the diet and feces, respectively, and Nd (g/kg) and Nf (g/kg) represent the nutrients in the diet and feces, respectively.

### 3.4. Milk Collection and Analysis

In the last 3 d of the pre-experiment period, and in periods 1 and 3, milk yield and milk samples from each cow were recorded and collected. Milk samples were collected at each milking time and then mixed together based on milk yield. A total of 50 mL of mixed milk samples were collected to determine milk components, and milk urea nitrogen (MUN) was determined. Milk protein, milk non-fat solids, milk lactose and milk fat were determined by the milk analyzers (Lactoscan LAW, Milkotronic Ltd., Nova Zagora, Bulgaria). MUN were determined by a kit purchased from Nanjing Jiancheng Bioengineering Institute (Nanjing, China), according to the manufacturer’s instructions.

### 3.5. Fecal Samples Collection and Analysis

Before feeding on the last day of the pre-experiment period, and periods 1 and 3, fecal samples were collected by rectal palpation, 10 g fresh feces were mixed 1:2 with distilled water to measure pH with a pH meter (FE28-Standard, Beijing, China). A total of 15 g of fecal samples were preserved at −20 °C to determine the content of volatile fatty acids (VFA) and ammoniacal nitrogen (NH_3_-N). NH_3_-N was determined using the colorimetric method [18]. Fecal VFA concentrations were determined using the method by [19] with an Agilent 6890B Gas chromatograph (Agilent, Guangzhou, China) with HP-INNOwax capillary column (30.0 m × 320 μm × 0.5 μm) and FID detector. The parameters of the gas chromatograph were as follows: N_2_ as carrier gas, 40:1 splitting concentration, 0.4 μL and 220 °C injection volume and temperature, and 120 °C (3 min)–10 °C/min–180 °C (1 min) as the column chamber program. The composition of each sample was obtained using 2-ethylbutyric acid as the internal standard.

Extraction of total DNA from all fecal samples was conducted according to the instructions provided with the Omega Stool (Omega Bio-Tek, Norcross, GA, USA) kit. Selection of the V3-V4 region of the 16SrDNA gene was conducted by PCR amplification. The upstream primer sequence of the amplified region is 5′-CCTAYGGGRBGCASCAG-3′ and the downstream primer is 5′-GGACTACNNGGGTATCTAAT-3′. Gene libraries using kits (Pacific Biosciences, California, USA) was prepared from SMRTbellTM templates, and assessment of generation sequencing libraries quality was conducted using Qubit@2.0 fluorometers (Thermo Fisher, Waltham, MA, USA) and FEMTO Pulse systems (Agilent, Guangzhou, China). Sequencing of gene sequencing libraries was conducted using the Illumina NovaSeq platform (BIOTREE, Shanghai, China). PCR-free gene library construction and paired-end sequencing was conducted using the Illumina Nova sequencing platform (BIOTREE, Shanghai, China). A total of 89,879 valid data were obtained by splicing the reads and quality control. The 97% consistent sequences were clustered into OTUs (Operational Taxonomic Units) and the OTUs sequences were species annotated using the Silva138 database. The relative abundance of fecal microbiota at the level of phylum, family and genus were analysed using QIME (Version 1.8.0) software. Analysis of the alpha diversity of fecal microbiota was conducted using MOTHUR (Version 1.30) software; calculation of Unifrac distances was conducted using phyloseq script to measure beta diversity.

## 4. Statistical Analysis

This study was a completely randomized, cross-over design. Dry matter intake, milk yield and apparent nutrient digestibility were analysed using the MIXED program in SAS 9.4 (SAS Institute Inc, Cary, NC, USA) software, and the specific model is as follows:

Yijk = μ + Tk + Sj +Di+ Eijk

Yijk = value of the dependent variable; μ = overall mean; Tk = diet treatment effect; Sj = random effects of test cattle; Di = random effects of test period; and Eijk = random error. The results for all the data were listed as least square means, and were compared by Tukey’s test and were measured using a Spearman’s correlation rank. When *p* < 0.05, it was considered statistically significant, and 0.05 < *p* < 0.1 was considered a significant trend.

## 5. Results

### 5.1. DMI, Milk Yield and Milk Components

DMI and milk performance are shown in Table 3. Compared with the cows fed the CON diet, cows fed the IS and IO diet had lower milk fat concentrations, energy corrected milk (ECM), 3.5% fat corrected milk (FCM) yield and feed efficiency (*p* < 0.05); however, milk yield and DMI were not different among the three dietary treatments. Cows fed the IO diet had lower milk fat concentrations than those in the IS group (*p* < 0.001). However, there were no significant differences in the milk concentrations of protein, lactose and urea nitrogen among the three dietary treatments. Cows fed the IO diet had a lower milk fat yield than those in the CON group (*p =* 0.022), and cows fed the IS diet showed a trend for decreasing milk fat yield compared to cows fed the CON diet (*p =* 0.069). Compared with cows fed the IO diet, the milk protein yield of cows fed the IS diet tended to decrease (*p =* 0.078), and the yield of other milk components were not different among the three dietary treatments.

### 5.2. Apparent Nutrient Digestibility

The AOAC standard was used to determine the corresponding nutrients in diets and feces, and the results are shown in Table 4. Compared with the cows fed the CON diet, the cows fed the IS diet had a higher intake of starch (*p* < 0.001), and a lower intake of ADF (*p* = 0.004), digestibility of NDF (*p* = 0.004), ADF (*p* < 0.001) and EE (*p* < 0.001); the cows fed the IO diet had a higher intake of EE (*p* < 0.001), and had a lower intake of NDF (*p* = 0.014) and ADF (*p* = 0.004), digestibility of NDF (*p* = 0.004), ADF (*p* = 0.001) and EE (*p* = 0.001). Compared with the cows fed the IS diet, the cows fed the IO diet had a higher intake of EE (*p* < 0.001) and had a lower intake of starch (*p* < 0.001) and digestibility of EE (*p* = 0.001). However, the intake and digestibility of DM, OM and CP were not different among dietary treatments.

### 5.3. Fecal Fermentation Parameters Effectiveness of Different Diet Treatments

As shown in Table 5, the cows fed the IS diet had a lower fecal pH than cows fed the CON diet and the IO diet (*p* < 0.05), and the cows fed the IS diet and IO diet had a lower NH_3_-N concentration than cows fed the CON diet (*p* < 0.05). Cows fed the IS and IO diet had higher levels of acetate and propionate than cows fed the CON diet (*p* < 0.05). Cows fed the IS diet had a lower acetate to propionate ratio than cows fed the CON diet (*p* < 0.05). In addition, compared to the cows fed IO diet, cows fed the IS diet had lower levels of valerate (*p* < 0.05).

### 5.4. Feces Bacteria Abundance Effectiveness of Different Diet Treatments

**Figure 1:**
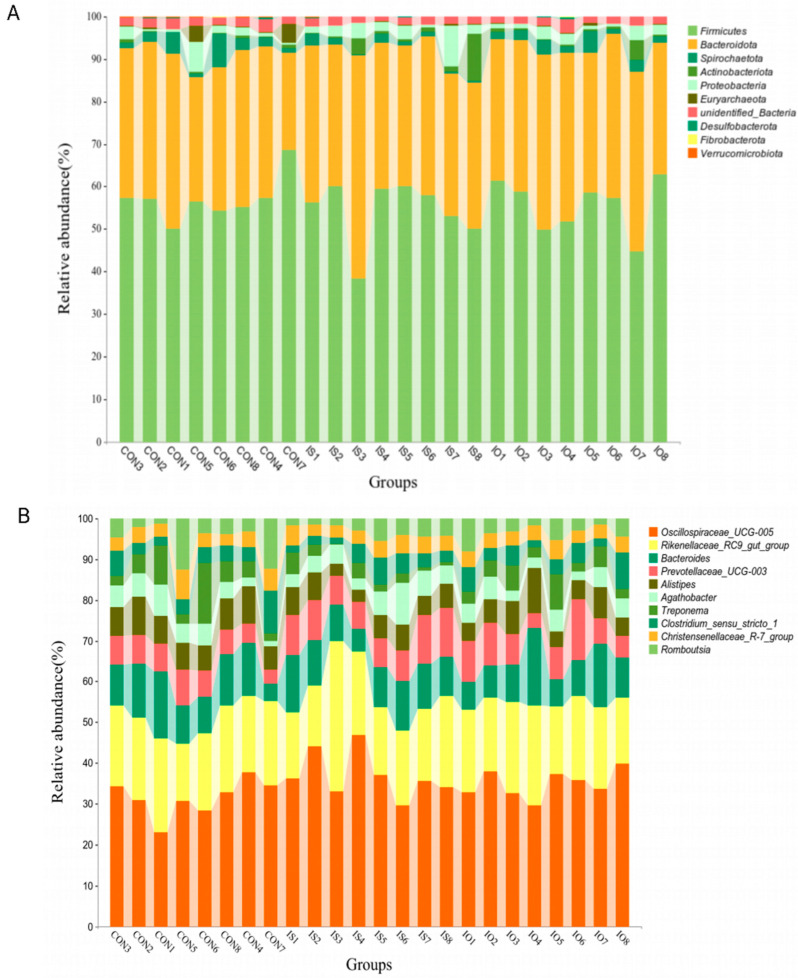
(**A**). Effect of dietary treatment on accumulation map of feces microflora at the phylum; (**B**). Effect of dietary treatment on accumulation map of feces microflora at the genus.

**Figure 2:**
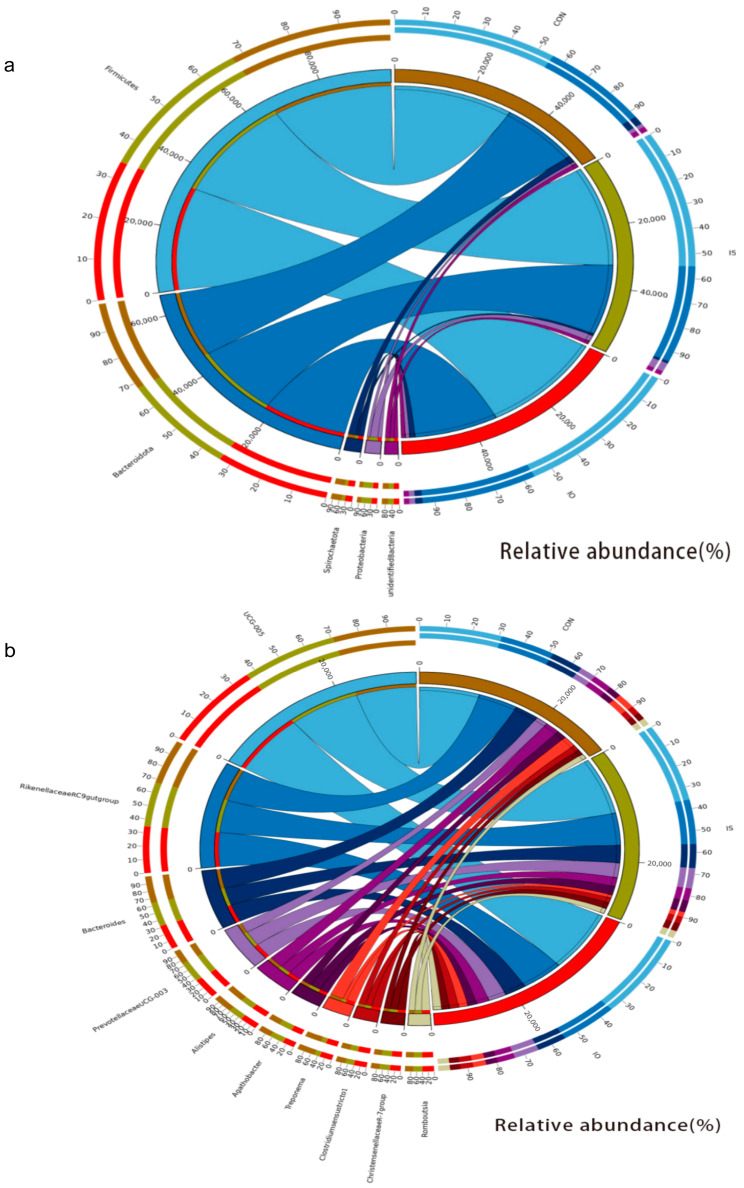

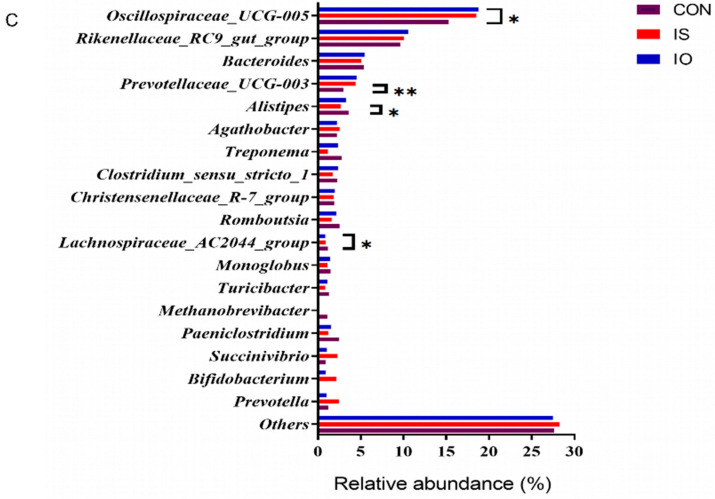
Circos map of feces microflora at the phylum (**a**) and genus (**b**) levels for test cows; (**c**) Effects of different dietary treatments on the feces microflora (genus level; relative abundance >1%; * *p* < 0.05, ** *p* < 0.01) (CON = fed the control diet; IS = fed the high-starch diet; IO = fed the high-fat diet; and number = cow number).

A total of 31 phyla and 419 genera were identified in the feces by taxonomic analysis. and lists the top 10 bacterial phyla and genera (Figure 1A,B). As shown in Figure 2a and Table 6. Firmicutes (56.12% vs. 53.61% vs. 54.89%) and Bacteroidota (33.40% vs. 36.41% vs. 36.29%) were the 2 dominant phyla in the CON, IS and IO groups, respectively. We observed the relative abundance of Verrucomicrobiota was lower in the IS and IO cows than in the CON cows (*p* < 0.01).

At the genus level, 419 genera were identified. We only listed the 18 bacterial genera whose relative abundance was higher than 1% in at least one group. As shown in Figure 2b, the cows fed the CON, IS and IO diet were dominated by Oscillospiraceae UCG-005 (15.28% vs. 18.54% vs. 18.76%) and Rikenellaceae_RC9_gut_group (9.67% vs. 10.06% vs. 10.56%). According to Table 7 and Figure 2c, we observed that the cows fed the CON diet had a lower Oscillospiraceae_UCG-005 relative abundance, out of the three types of rations, and the cows fed the CON diet had a higher relative abundance of Lachnospiraceae_AC2044_group (1.18% vs. 0.86%) than the IO diet, while the IS cows tended to have less Lachnospiraceae_AC2044_group than those on the CON diet (*p* = 0.053). In addition, compared with CON cows, the relative abundance of PrevotellaceaeUCG-003 in IS cows was increased by 47.32% (*p* = 0.004) and the relative abundance of Alistipes in IS cows was 26.33% lower (*p* = 0.031).

### 5.5. Diversity of Fecal Microbial Communities

**Figure 3:**
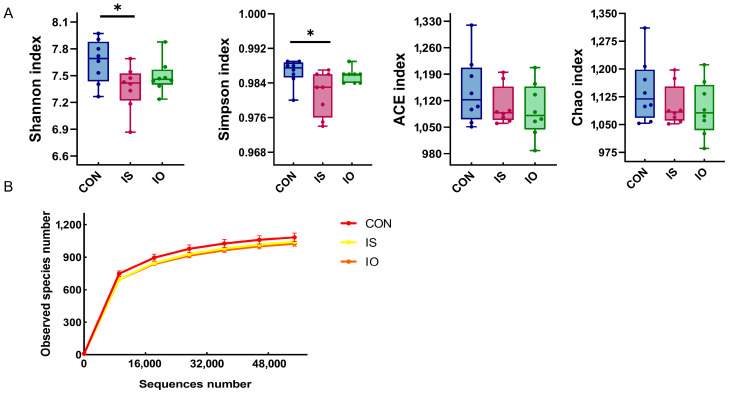
Effect of different dietary treatments on the feces microflora of dairy cows. (**A**) Alpha-diversity of the different dietary treatments (* *p* < 0.05); (**B**) rarefaction curve of the different dietary treatments; CON = fed the control diet; IS = fed the high-starch diet; IO = fed the high-fat diet; and number = cow number.

**Figure 4:**
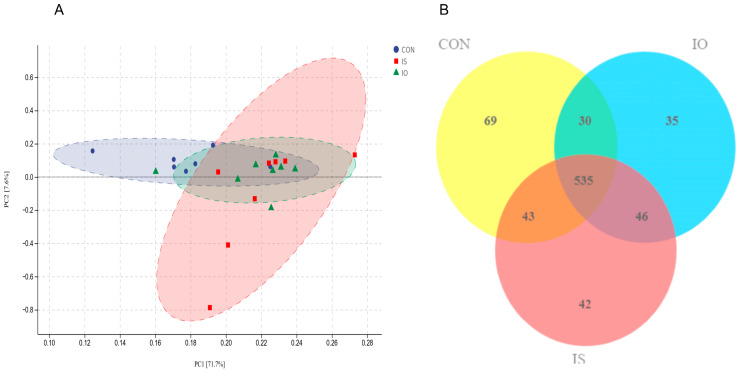
Effect of different dietary treatments on the feces microflora of dairy cows. (**A**) Principal coordinates analysis (PCoA) of different diet treatments and (**B**) Venn diagram of the OTUs of different diet treatments.

The alpha diversity, species accumulation curves, OTUs Venn and PCA chart of the fecal bacterial community for the different dietary treatments are shown in Figure 3 and Figure 4. In this study (Figure 3A,B), with the increase in sample sizes, the species accumulation curve tended to a plateau, showing that the test sample was sufficient and met the requirements of the test and that the results of further analysis were reliable, and there were no differences in the performance of the Shannon, Simpson, Chao and ACE indices between the different dietary treatments, except for the Simpson and Shonnon indices were higher in the CON cows than in the IS cows. As shown in Figure 4B, 535 OTUs were present in each treatment, and they could be regarded as core microflora; 69, 35 and 42 OTUs were unique to CON, IO and IS groups, respectively.

### 5.6. Correlation of Feces Bacterial Differentiation with Milk Components and Nutrient Digestibility

**Figure 5:**
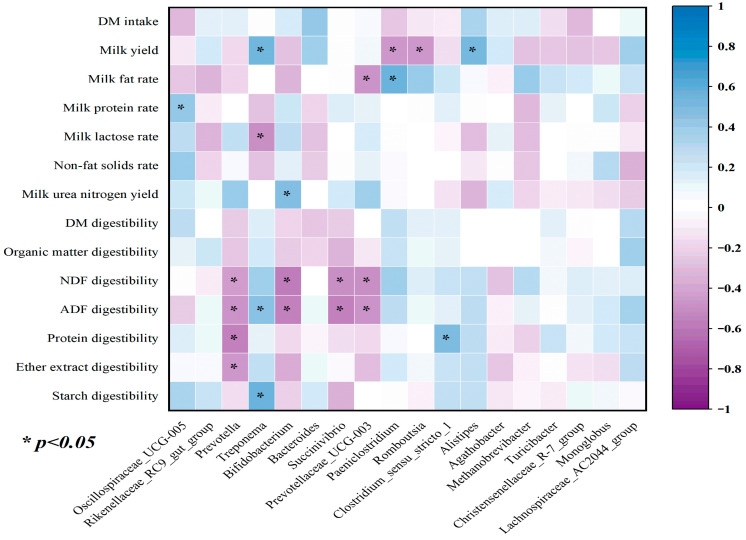
The correlations between the differential bacteria in different dietary treatments and the milk components and nutrient digestion. Violet color indicates negative correlations and blue color represents positive correlations. Color darkness stands for the value of correlation coefficients, the darker the color, the greater the coefficient. The asterisk indicates the correlation is statistically significant (*p* < 0.05). (CON = fed the control diet; IS = fed the high-starch diet; and IO = fed the high-fat diet. DM = dry matter; ADF = acid detergent fiber; and NDF = neutral detergent fiber.).

The Spearman’s rank correlation analysis was used to explore the correlations between milk components (%), nutrient digestibility and the relative abundances of those bacterial genera that differed significantly between cows fed the CON, IS and IO diet. The results are shown in Figure 5. The milk fat percentage was negatively correlated with the abundances of Prevotellaceae_UCG-003, and Paeniclostridium was correlated positively with milk fat percentage. In addition, the abundances of Prevotella, Succinivibrio, Bifidobacterium and Prevotellaceae_UCG-003 correlated negatively with ADF and NDF digestibility. The relative abundance of Treponema and Prevotella had positive and negative correlations with ADF and starch digestibility, respectively.

## 6. Discussion

### 6.1. DMI, Milk Yield and Milk Components

MFD was a syndrome characterized by a reduction in milk fat concentration [3]. In this study, milk fat concentration and yield in cows fed the IS and IO diets were consistent with cows in the MFD condition. In this trial, DMI and milk yield did not differ significantly between different dietary treatments; these results are similar to other studies [21,22]. Milk fat yield and concentration were significantly decreased, while the rest of milk components and yield did not change significantly, similar to what some other researchers have found [23,24]. The reduction of milk fat concentration and yield may be due to lower pH and higher propionate levels in the hindgut [3]. The fermentation pattern of the rumen was altered by the lower pH in cows [25] and may have increased rumen occurrence of biohydrogenation intermediates such as trans-10, cis-12 and trans-9. High levels of propionate can be produced by high-starch diets, which can inhibit the transfer of precursors required for milk fat synthesis to the mammary gland [26]. In addition, propionate decreased all the even-chain FA uniformly, regardless of the length of their carbon chain, and increased the percentage of odd-chain FA [5]. This may be one of the reasons why high-fat diets reduced milk fat yield and concentration in cows. Meanwhile, the rumen bio-hydrogenation (BH) pathway in cows could be directly modified by a high-fat diet through incomplete hydrogenation of polyunsaturated fatty acids and, as a result, the milk fat concentration was reduced in the cows [27]. Moreover, SARA could be induced by low runimal pH [28].

### 6.2. Apparent Nutrient Digestibility

In this trial, cows fed the IS diet had a lower ADF and NDF digestibility. This result may be due to the higher rumen fermentation rate of finely crushed wheat, as fermentable carbohydrate content was usually negatively correlated with rumen fiber degradation rate [29]. In addition, rumen VFA production was increased by a high-starch diet, which reduced pH when VFA exceeded rumen saturation, and fiber digestibility was reduced [30]. While some researchers have found that the high-starch diet had lower NDF digestibility [31]. Using the in-situ nylon bag technique, the researcher found that when cows were exposed to SARA-induced pH reduction, NDF degradation rates of hay in situ decreased from 31.5% and 51.3% to 24.6% and 36.9% at 24 h and 48 h, respectively [31]. Since the rumen was the main point of nutrient digestion, such as fiber, when rumen digestibility of nutrients was reduced, whole gut digestibility performance was reduced synchronously.

In our study, the digestibility of ether extract was decreased except that the fiber digestibility was decreased by the high-fat diet, which is similar to the results of previous studies [32]. This may be due to the fact that fiber-degrading bacteria were inhibited by unsaturated fatty acids in the high-fat diet [33]. Meanwhile, studies have shown that the hydrophobic and amphiphilic nature of the fat has a negative effect on the digestion and fermentation of nutrients in rumen [34,35].

### 6.3. Fecal Fermentation Parameters

In Table 5, lower fecal pH in cows fed the IS diet was caused by the higher yield of short-chain fatty acids from high-starch diets [36]. A previous study has shown that fecal pH is not affected by high-fat diets [37], similar to the results of our study. We observed that the two induction groups had lower fecal NH_3_-N concentrations, because the fecal NH_3_-N was reduced by a high-starch diet and lower pH [38], and high-fat diets can inhibit microbial protein synthesis pass direct disruption of microbial cell membrane and cellular function by the unsaturated fatty acids, and reduce fecal NH_3_-N concentrations, altering the microbiota by inhibiting protozoan populations [39].

In our study, the cows fed with IS and IO diets had higher concentrations of acetate. As one of the main precursors of milk fat synthesis, acetate regulated milk fat synthesis and gene expression [40]. One possibility was that shortage of glucose supply may be the limiting factor of milk fat synthesis when acetate concentration is too high [41]. The ratio of acetate to propionate was decreased, which was consistent with the previous study [42]. In this study, cows fed with the IS and IO diets had higher concentrations of propionate; additional starch in the diet can be degraded by starch-degrading bacteria and increased propionate concentrations [43]. Moreover, triacylglycerols in high-fat diets can be hydrolyzed to produce propionate [44]. Meanwhile, in the rumen, increased hydrogen due to inhibition of methanogens by PUFA can cause excess reduced nicotinamide adenine dinucleotide to be transported for propionate production [45], which may further increase whole gut propionate. The cows fed with the IO diet had a higher concentrations of valerate, which was related to their higher starch digestibility [46], and had a higher isobutyrate and butyrate than the other cows, but it was not significant, similar to the results of previous researchers [47,48].

### 6.4. Fecal Bacteria Abundance

Alpha diversity indicates the diversity and abundance of microflora in a sample, and the changes were inversely proportional to the intestinal pathogen [49]. As can be seen from Figure 3, that the cows fed the IO diet had a lower Simpson and Shannon index compared with the cows fed the CON diet, suggesting that the high-starch diet resulted in an imbalance of hindgut microbiota [50]. The Firmicutes and Bacteroidota were considered by previous researchers to be the majority of the dominant gut microbiota in various mammals and to play an important role in the gut microbial ecology [51]. From Table 6 and the circos map (Figure 2a), Firmicutes and Bacteroidota were not significantly different among CON, IO and IS cows, indicating that the dominant microbial phylum was less affected by the high-fat and high-starch diets. The Spirochaetota acted mainly on the hydrolysis of complex polysaccharides in the plant cell wall, the degradation of proteins and the production of B vitamins in the rumen [52], and this may be one of the reasons for the lower protein and fiber digestibility in the cows fed the IS diet. Verrucomicrobiota was significantly lower in the cows fed the IO and IS diets than in the cows fed the CON diet, which played an important role in animal immune regulation and intestinal health, and its abundance was positively correlated with immunity [53], indicating that high-fat and high-starch diets reduced hindgut immunity.

As shown in Table 7 and Figure 2C, one remarkable alteration in the present study was the increased relative abundance of Oscillospiraceae UCG-005 in the cows fed the IO diet, which can use host glycans as growth stimulants. Some researchers have shown that relative abundance of Oscillospiraceae UCG-005 plays a positive role in the intestinal health of animals [54,55]. Meanwhile, we observed a higher relative abundance of Prevotellaceae_UCG-003 in IS and IO cows, which raised the potential for enteritis [56], and the change may be related to the ruminal BH pathway; but the mechanism of action was unclear [57]. At the same time, the cows fed the IO diet had a lower relative abundance of Alistipes, similar to previous results [58], and was attributed to the reduced fibrous substrate of IS cows feeding. Treponema is a known fiber degrader and was positively correlated with fiber degradation [59], which was also one of the reasons for the lower fiber degradation in the cows fed the IS diet. Similarly, the decrease in Lachnospiraceae_AC2044_group in the cows fed the IO and IS diet may also be associated with the decrease in NH_3_-N reported in our companion study [60], and it has been reported to be negatively correlated with branched-chain volatile fatty acids in yaks grazing on low-CP (10%) and high-NDF shrubs [61].

In addition, our research showed an interesting result that the abundances of Prevotella, Bifidobacterium, Succinivibrio and Prevotellaceae_UCG-003 negatively correlated with the digestion of ADF and NDF, which explained the lower proportion of ADF and NDF digestibility in the cows fed the IS and IO diet. Paeniclostridium was reportedly one of the two largest genera in heifers of the Holstein-Fresian breed in another study and were correlated to their digestive functions [62], which explained the greater digestibility of ADF and NDF and the milk fat concentration in the cows fed the CON diet. Furthermore, the analysis of PCoA and bacterial abundance confirmed that IO and IS diets play a pronounced role in the correlation analysis. However, the exact mechanism remains unclear, thus requiring further studies.

## 7. Conclusions

In this trial, both a high-starch and a high-fat diet can result in MFD in dairy cows, resulting in significantly lower milk fat percentage, milk fat yield, 3.5% FCM and ECM yield, as well as reduced digestibility of ADF and NDF. Additionally, the high-fat diet reduced the digestibility of ether extracts in dairy cows. Both a high-starch and a high-fat diet can reduce the concentrations of NH_3_-N and increase the concentrations of propionate and acetate in feces. A high-fat diet can increase the concentration of valerate, and high-starch diets can decrease pH in feces. In addition, we observed changes in the hindgut flora of cows, suggesting that hindgut flora may also be involved in MFD in cows, but this relationship needs to be further investigated by looking at the relationship between hindgut flora and trans fatty acids in cows.

## Figures and Tables

**Table 1:** Composition and nutritional levels of experimental diets.

Ingredients (% of DM ^2^)	Diet ^1^, %		
	CON	IS	IO
Whole corn silage	25.55	17.42	25.07
Alfalfa hay	15.26	10.38	14.97
Oat hay	6.28	4.25	6.18
Ground corn	13.27	13.27	13.02
Steam-flaked corn	6.02	6.02	5.91
Fine ground wheat	-	15.01	-
Sunflower oil	-	-	5.86
Soybean meal	15.42	14.09	17.01
Sunflower seed meal	0.73	0.73	0.71
Extruded soybean	0.84	0.84	0.82
Sugar beet pulp	1.68	1.68	-
Soybean hull	4.29	5.65	-
Whole cottonseed	6.14	6.14	6.02
Cane molasses	1.55	1.55	1.53
Mineral–vitamin premix ^3^	0.42	0.42	0.42
Dicalcium phosphate	0.43	0.43	0.42
Limestone	0.87	0.87	0.85
Sodium bicarbonate	0.75	0.75	0.73
Magnesium oxide	0.22	0.22	0.21
Salt	0.28	0.28	0.27
Nutritional levels ^4^, % DM			
OM	93.71	94.43	93.95
CP	16.47	16.52	16.52
NE_L_, MJ/kg ^5^	6.71	7.03	7.70
NDF	32.17	30.41	28.31
ADF	15.84	13.84	13.15
Starch	23.51	31.93	22.97
EE	2.60	2.53	8.13
Calcium	0.87	0.79	0.82
Total phosphorus	0.40	0.41	0.40

^1^ CON = basic total mixed ration; IS = high-starch diet with crushed wheat; IO = high-fat diet with sunflower fat. ^2^ DM = dry matter. ^3^ Premix, per kg (DM basis) contained: Vitamin A, 1,000,000 IU; Vitamin D, 392,000 IU; Vitamin E, 10,080 IU; Iodine, 120 mg; Selenium, 66 mg; Cobalt, 100 mg; Copper, 2000 mg; Zinc, 18,000 mg; and Manganese, 11,000 mg. ^4^ Nutritional levels: OM = organic matter; CP = crude protein; ADF = acid detergent fiber; NDF = neutral detergent fiber; EE = ether extract. ^5^ Calculated using NEL values of feedstuffs from Nutrient Requirements of Dairy Cattle (NRC, 2001)

**Table 2:** Treatment assignment of a cross-over design to study the effect of dietary starch or fat on milk fat depression ^1^.

Assignment	Pre-Experiment	Period 1	Period 2	Period 3
1	CON	IS	Wash period	IO
2	CON	IO	Wash period	IS

^1^ CON/Wash period = basic total mixed ration; IS = high-starch diet; and IO = high-fat diet.

**Table 3:** Effect of dietary treatment on DMI and milk performance of dairy cows.

Items ^1^	Treatments ^2^			SEM	*p*-Value		
	CON	IS	IO		C vs. IS	C vs. IO	IS vs. IO
DMI, kg/d	22.79	22.83	23.12	0.286	0.999	0.913	0.917
Milk, kg/d	29.52	28.26	30.92	0.509	0.601	0.534	0.065
Milk components, %							
Milk fat	3.96 ^a^	3.26 ^b^	2.69 ^c^	0.115	<0.001	<0.001	<0.001
Milk protein	3.11	3.16	3.16	0.018	0.284	0.316	0.997
Milk lactose	4.72	4.84	4.82	0.025	0.150	0.206	0.979
Yield, kg/d							
Milk fat	1.17 ^a^	0.91	0.82 ^b^	0.037	0.069	0.022	0.363
Milk protein	0.92	0.89	0.98	0.016	0.927	0.642	0.078
Milk lactose	1.39	1.37	1.49	0.026	0.893	0.213	0.101
3.5%FCM	31.68 ^a^	26.72 ^b^	26.72 ^b^	0.633	0.004	0.004	1.000
ECM	31.36 ^a^	27.25 ^b^	27.80 ^b^	0.526	0.002	0.006	0.829
Milk urea nitrogen, mg/dL	17.06	19.25	20.42	0.821	0.419	0.153	0.772
Feed efficiency ^3^	1.38 ^a^	1.19 ^b^	1.21 ^b^	0.025	0.005	0.007	0.977

^1^ DMI = DM intake; 3.5% FCM = 3.5% fat corrected milk, calculated with the formula: 16.23 × milk fat production + 0.4318 × milk yield; ECM = energy corrected milk, calculated with the formula: 7.2 × milk protein yield + 0.327 × milk yield + 12.95 × milk fat yield. ^2^ CON/C = control group; IS = high-starch diet group; IO = high-fat diet group. ^3^ Feed efficiency = kg of ECM/kg of DMI [20]. ^a,b,c^ Different letters indicate significant differences (*p* < 0.05).

**Table 4:** Effect of dietary treatment on nutrient digestibility in dairy cows.

Items ^1^	Treatments ^2^			SEM	*p*-Value		
	CON	IS	IO		C vs. IS	C vs. IO	IS vs. IO
Nutrient intake, kg/d							
DM	22.79	22.83	23.12	0.286	0.999	0.913	0.917
OM	21.69	20.94	20.45	0.231	0.260	0.042	0.543
CP	3.74	3.93	3.98	0.061	0.422	0.269	0.941
NDF	7.27	6.94	6.55	0.104	0.295	0.014	0.215
ADF	3.45 ^a^	3.16 ^b^	3.04 ^b^	0.046	0.034	0.004	0.224
EE	0.85 ^b^	0.86 ^b^	2.01 ^a^	0.115	0.932	<0.001	<0.001
Starch	4.86 ^b^	7.16 ^a^	5.18 ^b^	0.227	<0.001	0.473	<0.001
Nutrient digestibility, %							
DM	75.48	73.47	73.67	0.403	0.108	0.154	0.975
OM	74.83	72.72	72.87	0.410	0.091	0.122	0.984
CP	66.89	64.49	69.56	1.252	0.710	0.658	0.248
NDF	55.06 ^a^	46.50 ^b^	47.93 ^b^	1.064	<0.001	0.004	0.700
ADF	60.05 ^a^	49.30 ^b^	51.15 ^b^	1.228	<0.001	0.001	0.605
EE	81.73 ^a^	79.43 ^a^	70.16 ^b^	1.332	0.597	0.001	0.001
Starch	92.85	92.31	93.46	0.540	0.917	0.897	0.683

^1^ DMI = DM intake; OM = organic matter; CP = crude protein; NDF = neutral detergent fibers; ADF = acid detergent fibers; EE = ether extract. ^2^ CON/C = control group; IS = high-starch diet group; IO = high-fat diet group. ^a,b^ Different letters indicate significant differences (*p* < 0.05).

**Table 5:** Effect of dietary treatment on hindgut fermentation parameters of dairy cows.

Items ^1^	Treatments ^2^			SEM	*p*-Value		
	CON	IS	IO		C vs. IS	C vs. IO	IS vs. IO
pH	6.76	6.59	6.75	0.023	0.025	0.969	0.038
NH_3_-N, mg/100 mL	3.53 ^a^	2.96 ^b^	3.04 ^b^	0.067	0.011	0.028	0.689
Acetate, mmol/L	14.86 ^b^	18.51 ^a^	18.03 ^a^	0.442	0.023	0.049	0.741
Propionate, mmol/L	2.93 ^b^	3.86 ^a^	4.23 ^a^	0.131	0.004	0.033	0.061
Valerate, mmol/L	0.22 ^b^	0.20 ^b^	0.27 ^a^	0.011	0.613	0.049	0.008
Butyrate, mmol/L	2.80	3.18	3.29	0.118	0.260	0.116	0.876
Isobutyrate, mmol/L	0.19	0.19	0.25	0.013	1.000	0.128	0.128
Acetate: Propionate	5.12 ^a^	4.39 ^b^	4.69 ^ab^	0.105	0.013	0.155	0.369
TVFA	21.00	25.72	25.91	0.626	0.079	0.095	0.9771

^1^ pH = potential of hydrogen; NH_3_-N = ammoniacal nitrogen; TVFA = total volatile fatty acid. ^2^ CON/C = control group; IS = high-starch diet group; IO = high-fat diet group. ^a,b^ Different letters indicate significant differences (*p* < 0.05).

**Table 6:** Effect of dietary treatment on feces microflora of dairy cows (phylum levels, %).

Items	Treatments ^1^			SEM	*p*-Value		
	CON	IS	IO		C vs. IS	C vs. IO	IS vs. IO
Firmicutes	56.12	53.61	54.89	1.258	0.447	0.677	0.712
Bacteroidota	33.40	36.41	36.29	1.097	0.332	0.249	0.966
Spirochaetota	2.84	1.16	2.35	0.354	0.084	0.619	0.050
Actinobacteriota	0.44	2.33	1.01	0.479	0.172	0.310	0.363
Proteobacteria	1.96	3.13	1.84	0.424	0.355	0.894	0.224
Euryarchaeota	1.18	0.15	0.20	0.235	0.143	0.162	0.530
unidentified_Bacteria	2.11	1.64	1.82	0.096	0.037	0.262	0.416
Desulfobacterota	0.11	0.08	0.10	0.018	0.419	0.935	0.504
Fibrobacterota	0.01	0.01	0.04	0.012	0.820	0.447	0.407
Verrucomicrobiota	0.17 ^a^	0.03 ^b^	0.02 ^b^	0.018	0.003	<0.001	0.442

^1^ CON/C = control group; IS = high-starch diet group; IO = high-fat diet group. ^a,b^ Different letters indicate significant differences (*p* < 0.05).

**Table 7:** Effect of dietary treatment on feces microflora of dairy cows (genus levels, %).

Items	Treatments ^1^			SEM	*p*-Value		
	CON	IS	IO		C vs. IS	C vs. IO	IS vs. IO
Oscillospiraceae_UCG-005	15.28 ^b^	18.54	18.76 ^a^	0.751	0.104	0.028	0.905
Rikenellaceae_RC9_gut_group	9.67	10.06	10.56	0.468	0.783	0.311	0.695
Prevotella	1.20	2.44	1.01	0.570	0.486	0.813	0.373
Treponema	2.81	1.16	2.31	0.354	0.088	0.612	0.058
Bifidobacterium	0.20	2.16	0.90	0.479	0.155	0.218	0.385
Bacteroides	5.34	5.07	5.44	0.335	0.744	0.923	0.642
Succinivibrio	0.91	2.29	1.03	0.364	0.203	0.770	0.237
Prevotellaceae_UCG-003	2.98 ^b^	4.39 ^a^	4.52	0.307	0.004	0.069	0.879
Paeniclostridium	2.46	1.21	1.50	0.290	0.131	0.256	0.446
Romboutsia	2.53	1.60	2.12	0.277	0.213	0.615	0.324
Alistipes	3.57 ^a^	2.63 ^b^	3.25	0.190	0.031	0.484	0.201
Clostridium_sensu_stricto_1	2.23	1.70	2.37	0.252	0.395	0.834	0.270
Agathobacter	2.21	2.50	2.20	0.188	0.580	0.997	0.525
Methanobrevibacter	1.09	0.14	0.18	0.217	0.142	0.161	0.515
Turicibacter	1.29	0.84	1.09	0.141	0.206	0.627	0.397
Christensenellaceae_R-7_group	1.92	1.87	1.98	0.075	0.815	0.779	0.544
Monoglobus	1.48	1.11	1.46	0.083	0.096	0.919	0.055
Lachnospiraceae_AC2044_group	1.18 ^a^	0.90	0.86 ^b^	0.054	0.053	0.027	0.651
Others	27.64	28.26	27.46	1.079	0.831	0.954	0.745

^1^ CON/C = control group; IS = high-starch diet group; IO = high-fat diet group. ^a,b^ Different letters indicate significant differences (*p* < 0.05).

## Data Availability

The data analyzed during the current study are available from the corresponding author upon reasonable request.

## Abbreviations

MFD: milk fat depression; UFA: unsaturated fatty acids; BHBA: β-hydroxybutyric acid; TMR: total mixed ration; DMI: dry matter intake; NDF: neutral detergent fiber; ADF: acid detergent fiber; AIA: acid insoluble ash; MUN: milk urea nitrogen; VFA: volatile fatty acids; NH_3_-N: ammoniacal nitrogen; ECM: energy corrected milk; FCM: fat corrected milk; BH: rumen bio-hydrogenation; OM: organic matter; CP: crude protein; EE: ethanol extract.
